# Compartmentalized metabolic network reconstruction of microbial communities to determine the effect of agricultural intervention on soils

**DOI:** 10.1371/journal.pone.0181826

**Published:** 2017-08-02

**Authors:** María Camila Alvarez-Silva, Astrid Catalina Álvarez-Yela, Fabio Gómez-Cano, María Mercedes Zambrano, Johana Husserl, Giovanna Danies, Silvia Restrepo, Andrés Fernando González-Barrios

**Affiliations:** 1 Grupo de Diseño de Productos y Procesos (GDPP), Department of Chemical Engineering, Universidad de los Andes, Bogotá, Colombia; 2 Laboratorio de Micología y Fitopatología (LAMFU), Department of Biological Sciences, Universidad de los Andes, Bogotá, Colombia; 3 Center for Genomics and Bioinformatics of Extreme Environments (Gebix), Bogotá, Colombia; 4 Corporación Corpogen Research Center, Bogotá, Colombia; 5 Centro de Investigaciones en Ingeniería Ambiental, Department of Environmental Engineering, Universidad de los Andes, Bogotá, Colombia; National Taiwan University, TAIWAN

## Abstract

Soil microbial communities are responsible for a wide range of ecological processes and have an important economic impact in agriculture. Determining the metabolic processes performed by microbial communities is crucial for understanding and managing ecosystem properties. Metagenomic approaches allow the elucidation of the main metabolic processes that determine the performance of microbial communities under different environmental conditions and perturbations. Here we present the first compartmentalized metabolic reconstruction at a metagenomics scale of a microbial ecosystem. This systematic approach conceives a meta-organism without boundaries between individual organisms and allows the *in silico* evaluation of the effect of agricultural intervention on soils at a metagenomics level. To characterize the microbial ecosystems, topological properties, taxonomic and metabolic profiles, as well as a Flux Balance Analysis (FBA) were considered. Furthermore, topological and optimization algorithms were implemented to carry out the curation of the models, to ensure the continuity of the fluxes between the metabolic pathways, and to confirm the metabolite exchange between subcellular compartments. The proposed models provide specific information about ecosystems that are generally overlooked in non-compartmentalized or non-curated networks, like the influence of transport reactions in the metabolic processes, especially the important effect on mitochondrial processes, as well as provide more accurate results of the fluxes used to optimize the metabolic processes within the microbial community.

## Introduction

Soil represents one of the most diverse and complex natural environments on Earth in terms of species diversity and community niches [1]. The soil microbial communities contain approximately 2,000 to 18,000 different genomes per gram of soil, harboring tens of thousands of Eukaryotic and Prokaryotic taxa [1]. This microbial diversity represents a reservoir of genetic information, with vast potential for the exploitation of environmental resources and the search of compounds with possible industrial application [2]. The soil microbial communities are involved in a wide range of ecological processes with important economic impacts. They play a key role in the regulation of global biogeochemical cycles, in soil fertility, plant health, and are responsible for most nutrient transformations on earth. These factors influence agricultural productivity, plant and animal diversity, and earth’s climate changes [3,4]. Thus, it is important to unravel the functions of the microbial communities, the effects of natural and anthropogenic perturbations on them, and their interactions with the soil ecosystem [5].

Metagenomic models of soil microbial ecosystems can provide information about the metabolic potential of a particular environment and how microbial ecosystems respond to environmental perturbations [6]. The major challenge in metagenomic studies is determining the relationship between microbial composition and the functional diversity of an ecosystem. This is mainly due to the high variability of metabolic functions present in microbial communities and the interdependent relationship that exists among their members. Therefore, in order to understand microbial ecosystems as a whole, and how the interaction between all biological entities determine the dynamics of the community, it is necessary to develop a compartmentalized metabolic model capable of representing all of the microorganisms that make up the community. Multi-compartmentalized metabolic models provide information about ecosystems that is generally underestimated in non-compartmentalized networks, providing information about the metabolic processes in specific organelles and about the effect of transport reactions between compartments and between the intra and extra-cellular space [7]. Furthermore, they provide more accurate results of the fluxes used to optimize a specific metabolic process of the ecosystems, allowing a qualitative and quantitative analysis of metabolic processes carried out by the microbial communities. These metabolic fluxes show the contributions of diverse metabolic pathways to the general functions of the ecosystem [8]. The extension of genome-scale flux balance models to a metagenomics-scale can explain the evolution and dynamics of metabolic processes in microbial communities and allow the *in silico* characterization of ecosystems under different environmental conditions [9].

Systems biology has made strong efforts to extend metabolic models from individual species to multiple organisms to understand how microorganisms interact with each other [10]. These models capture the interactions between individual organisms through artificial microbial ecosystems that allow modeling of these metabolic exchanges, which occur across transport reactions that connect individual microorganism to a common extracellular environmental compartment [11]. One of the most recent and important developments of synthetic metabolic large-scale reconstructions has been the construction of a representation of human metabolism, which allows the conversion of biological knowledge into a mathematical structure that can subsequently be used to construct predictive models [12].

In this study we present the first compartmentalized metabolic reconstruction at a metagenomics scale of a microbial ecosystem. From DNA sequencing data we provide a comprehensive image of a microbial community and show an inventory of the metabolic functions performed by the ecosystem. More specifically, we conducted a systematic analysis of the microbial ecosystem in rhizosphere soil from a National Park (*Parque Nacional Natural de los Nevados*) located in the Colombian Andes, which is considered a biodiversity hotspot that contains unique ecosystems currently at risk. This systematic approach allowed us to treat the microbial communities as a whole, integrating fundamental biological knowledge with metabolic flux patterns, to create a thorough image of how a microbial community operates. Furthermore, in this study we provide a refined curation of the metabolic networks, using topological and optimization algorithms to ensure the correct metabolic exchange between all sub-cellular compartments and the continuity of fluxes between all metabolic pathways. This approach allowed us to determine the effect of agricultural intervention on soils, through diversity, topological, and metabolic patterns, revealing the consequences of natural and anthropogenic perturbations. We were able to identify the challenges associated with the process of compartmentalization in terms of methodological steps and computational costs, to quantify the effect of agricultural intervention, and to investigate how compartmentalization of metabolites and reactions affects flux balance predictions.

## Materials and methods

**Fig 1:**
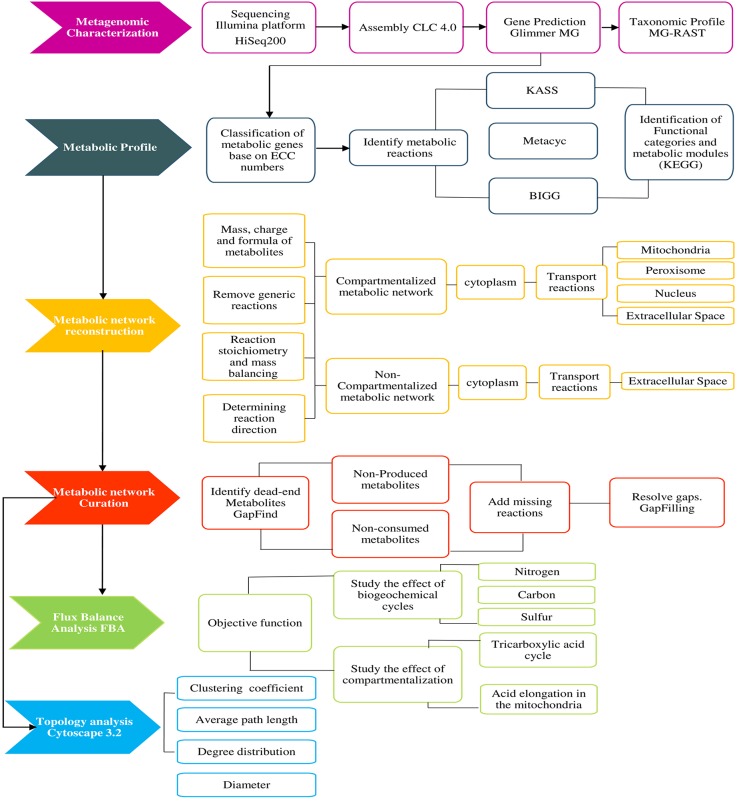
Pipeline used for the compartmentalized metabolic network analysis and functional reconstruction of the microbial community in agriculturally intervened and non-intervened soils.

The workflow used to carry out the reconstruction and characterization of a compartmentalized metabolic network at a metagenomics scale is shown in **Fig 1.**

### Metagenomic characterization

The Colombian Center for Genomics and Bioinformatics of Extreme Environments (Gebix) provided metagenomics sequencing data of two different soil samples collected from a Colombian Natural park (*Parque Natural de los Nevados*) located in the South American Andes mountains, a paramo ecosystem, above the continuous tree line and the permanent snow line. The first soil sample (S1) was collected at 3,677 meters above sea level from rhizosphere soil characterized by the following vegetation: *Cortaderia selloana*, *Pernettya prostrata*, *Buddleja* sp., *Lupinus albus*, and *Dendropanax* sp. This area is part of a protected area, and is not intervened by anthropogenic processes. The second soil sample (S2) was collected at 3,612 meters above sea level from the rhizosphere soil of a potato (*Solanum tuberosum*) field under conventional management conditions (application of chemicals such as fertilizers and pesticides). This represented the soil sample intervened by agricultural processes.

The samples were sequenced using the Illumina platform HiSeq2000 with paired-end reads. Sequences were filtered and trimmed based on length and quality. *De novo* assembly of the data was accomplished using the CLC Genomics Workbench version 4.0 [13] using the default parameters.

Contigs were analyzed using the gene predictor Glimmer MG (Gene Locator and Interpolated Markov Modeler—Metagenomics) [14], a platform for predicting genes from environmental DNA sequences. This algorithm selects the best of all possible combinations for predicting a gene based on an interpolated Markov model (MM) and identifies coding sequences with a sensitivity threshold of 99%.

The functional annotation was performed by searching for homologous relationships using KAAS (KEGG Automatic Annotation Server) [15] and MetaPathway [16]. The functional annotation of KAAS was performed with the BLAST tool (Basic Local Alignment Search Tool), taking Eukaryotes and Prokaryotes as representative data and using the assignment method BBH (bi-directional best hit). The functional annotation of MetaPathway was conducted using the BLAST tool against the MetaCyc database [17], using default parameters. The final functional annotation was the union of both results (annotations found in at least one of the methods).

Metabolic genes identified were classified based on the Enzyme Commission (EC) categories. Metabolic reactions catalyzed by the identified enzymes were found using biochemical and genetic databases such as KAAS [15], Metacyc [17], BiGG [18], and MetaPathway [16] (**S1 Table and S2 Table**). The metabolic functional profile was extracted using KAAS [15], which allows the identification of functional categories and metabolic modules reported in KEGG taking as a starting point the set of reactions that make up the metagenome.

To carry out the taxonomic analyses of the soil samples, we used the LCA (Lowest Common Ancestor) algorithm, implemented in MG-RAST [19]. This algorithm, calculates a taxonomic classification of the sequences in reference to the NCBI taxonomy database. Both metagenomics datasets were compared using default parameters: a maximum e-value of 1e^-5^, a minimum identity of 60%, and a minimum alignment length of 15 bp.

### Metabolic network reconstruction

A metabolic network reconstruction was performed based on the set of reactions identified in the annotation process. To construct refined metabolic networks (representing the main features of the microbial communities) we conducted the following four steps: *i]* Addition of information related to mass, charge, and formula, assuming an intracellular pH of 7.2 (for which data are reported) for each of the metabolites that made up a reaction (**S3 Table and S4 Table**). This information was obtained from the MetaNetX database [20]. *ii)* Removal of generic reactions that contain generic metabolites, i.e. metabolites that do not have specific stoichiometric coefficients, such as proteins, RNA, DNA, generic lipids, and glycan. *iii)* Reaction stoichiometry and balancing of mass and charge for each metabolite on both sides of the reaction. This was done using the MetaNetX database [20], which takes into consideration protons and water molecules that are often omitted in other biochemical databases. *iv)* To determine the directionality of the reactions, we extracted the values of the standard Gibbs free energy (Δ_*r*_*G*′) of each one of the reactions from the MetaCyc database [17]. For reactions lacking information in the MetaCyc database, we calculated the Δ_*r*_*G*′ by the method of group contribution proposed by [21]. Reaction directionally was assumed reversible when it was not possible to estimate the Δ_*r*_*G*′ (**S5 Table and S6 Table**).

### Metabolic network compartmentalization

To construct a compartmentalized metabolic network, reactions and proteins were assigned to specific cellular compartments. Incorrect assignment of the location of a reaction may lead to additional gaps in the metabolic network and misrepresentation of the network properties [22]. The prediction algorithm CELLO (Subcellular localization predictor) [23], was used to predict cellular localization based on characteristics of the amino acid sequences and taking Eukaryotic organisms as models of subcellular compartmentalization. The CELLO algorithm has two levels of a support vector machine. The classifiers of the first level are based on different combinations of the characteristics of the amino acid sequences such as physicochemical characteristics: polar, neutral, hydrophobic, acidic, basic, disulfide bridges, aliphatic, etc. The second level processes the outputs of the first level classifiers, to generate the probability of the distribution of the sub-cellular localization of a particular sequence [23]. In order to improve the predictions obtained with CELLO, we analyzed the compartmentalized reconstructions reported in MetaNetX [20] for organisms considered within our taxonomic profile (**S7 Table**).

Additional transport reactions were required to describe the exchange of compounds between the cellular compartments of the metabolic reconstruction, and the extracellular space and cytosolic compartment. This was required to ensure the exchange of metabolites among microbial communities and the environment. We carried out a process of data mining in databases such as BiGG [18] and Metacyc [17] to create a database of transport and exchange reactions. The set of reactions added to the models were those entirely composed by compounds belonging to the metabolic networks analyzed (**S8 Table**).

### Curation of the metabolic reconstructions

Given that the networks had a significant numbers of gaps, i.e., missing reactions and functions, dead-end metabolites were identified by using the algorithm of Gapfind developed by Kumar *et al*. [24]. Non-produced metabolites that is, metabolites that were not produced by any of the reactions or those that could not be imported through any of the existing uptake pathways were identified. Furthermore, the non-consumed metabolites, metabolites that are not consumed by any of the reactions in the network or those that were not exported by any of the secretion pathways were identified [24].

To resolve identified gaps we implemented the GapFilling optimization algorithm [24]. For this, we created a specific dataset based on the MetaNetX database [20] for each of the networks. Using these databases, reactions capable of solving the identified gaps were added to the metabolic networks. Curation of the compartmentalized models was considerably more difficult to do than for the non-compartmentalized networks. Curation of the compartmentalized model was performed in two steps: first, fluxes among inner compartments and the cytoplasm was restored and second, specific gaps for each compartment were resolved. The results from GapFind/GapFill introduce external metabolic reactions, which might affect the topological-properties and the final fluxes in FBA. However, these reactions might be associated to the large amounts of un-annotated sequences present in metagenomes.

### Metabolic characterization of the soil microbial communities

#### Flux balance analysis

In order to quantify the impact of the agricultural intervention on the soil samples and the effect of compartmentalization of the models, the mathematical representation of the metabolic networks was used to implement a FBA. To identify the flux distribution, we assumed that the metabolic network was optimized with respect to a certain objective function. The optimization problem is a linear programming (LP) problem; Objective functions are represented as follow [25] [8]:

$$Z=c^{T}∙v$$ (1)

Where *c* defines the coefficients or weights for each of the fluxes in *v*, *c* is a vector of zeros with the number 1 in the position corresponding to the objective function reaction. This general representation of *Z*, where the elements of *c* can be manipulated, enables the formulation of a number of diverse objective functions. The optimization strategy employed by the FBA attempts to find a solution *v* that optimizes *Z* [8] [26].

A canonical FBA calculation can be formally expressed as the following linear programing problem:

$$Maximize\;Z=\sum_{j=1}^{n}{c_{j}}^{T}v_{j}$$ (2)

$$Subject\;to\;\sum_{j=1}^{n}S_{ij}\;v_{j}=0\;\forall_{i}=1,\ldots ,m$$ (3)

$$v_{j}^{LB}\leq v_{j}\leq v_{j}^{UB}\;\forall_{j}=1,\ldots ,n$$ (4)

Where ***S*** is the stoichiometric matrix with ***m*** (metabolites) by ***n*** (reactions), ***v*** is the vector of metabolic fluxes, ***v***^***UB***^ is a vector of the upper bounds for all fluxes, ***v***^***LB***^ is a vector of the lower bounds for all fluxes and ***c*** is the vector defining the contribution of different fluxes to the objective function. To solve the linear problem, we used the General Algebraic Modeling System (GAMS), a high-level modeling system for mathematical programming problems. The values for upper and lower bounds were 100 and 0 mmol gDW^-1^ h^-1^, respectively, except for the reversible reactions in which the lower bounds were -1000 mmol gDW^-1^ h^-1^. These values were selected after varying the upper bound from 1000 to 100 mmol gDW^-1^ h^-1^, where we finally obtained an optimal solution that maximized the number of active fluxes.

#### Topology analysis of the metabolic networks

Topological analyses of the metabolic networks were carried out using the plugin Network Analysis [27] in Cytoscape 3.2 [28]. The main topological parameters evaluated were *i*) the clustering coefficient to evaluate the number of interactions among one metabolite and its neighbors and *ii*)the degree distributions to determine the number of metabolites directly connected to a given metabolite ***m***. In the evaluation of the networks, we differentiated between the in-degree distribution (when the edges target the metabolite ***m***) and the out-degree distribution (when the edges target the adjacent neighbors of ***m***) [29] (27)(27)(27). To analyze the compactness of the metabolic networks, we evaluated the average path length and the network diameter, which indicate the average and maximum number of links between all pairs of nodes, respectively [27] [29].

#### Principal component analysis

In order to determine the set of main metabolites in every sample a Principal Component Analysis (PCA) was conducted. A PCA, is a standard nonparametric statistical analysis with the main goal of finding patterns to reduce the dimensions in the dataset with minimal loss of information [30]. The PCA was conducted in R using the stoichiometric matrix of the metabolic networks.

## Results

### Agricultural intervention of soils leads to a loss in the metabolic capabilities of the ecosystem

As a result of the pre-processing and assembly of reads, 2,001,060 contigs were obtained for the non-intervened soil with an average length of 426 bp. For the agriculturally-intervened soil, a total of 1,485,172 contigs were obtained with an average length of 446 bp. After implementing Glimmer MG, a total of 2,227,616 and 1,660,101 protein-coding regions were obtained for the non-intervened and agriculturally-intervened soil samples, respectively.

The functional metabolic profile for the non-intervened sample resulted in a network that contained 2,334 metabolic reactions related to 1,617 unique enzymes. For the intervened soil sample, the resulting consensus network consisted of 2,082 metabolic reactions associated with 1,483 unique enzymes. We removed 213 and 66 reactions that contained generic or ambiguous compounds such as DNA, RNA, glycan, and lipids from the initial models for the non-intervened sample and for intervened sample, respectively (**Table 1**).

**Table 1:** Characteristics of the initial metabolic network reconstructions.

	S1	S2
**Total reads**	205,850,654	153,838,374
**Total Contigs**	2,001,060	1,485,172
**N50**	2,124	2,328
**Contig mean sequence length**	426 ± 433 bp	446 ± 569 bp
**Mean GC percent**	56 ± 7%	54 ± 9%
**Predicted Proteins**	2,064,635	1,565,352
**Initial reactions**	2,334	2,082
**Initial metabolites**	2,237	2,153
**Enzymes**	1,617	1,483
**Generic reactions removed**	213	66
**Compartmentalized reactions**	3,440	3,235
**Compartmentalized metabolites**	3,142	2,968
**Transport reactions**	740	740

S1: Non-intervened soil sample; S2: Agriculturally-intervened soil sample.

**Fig 2:**
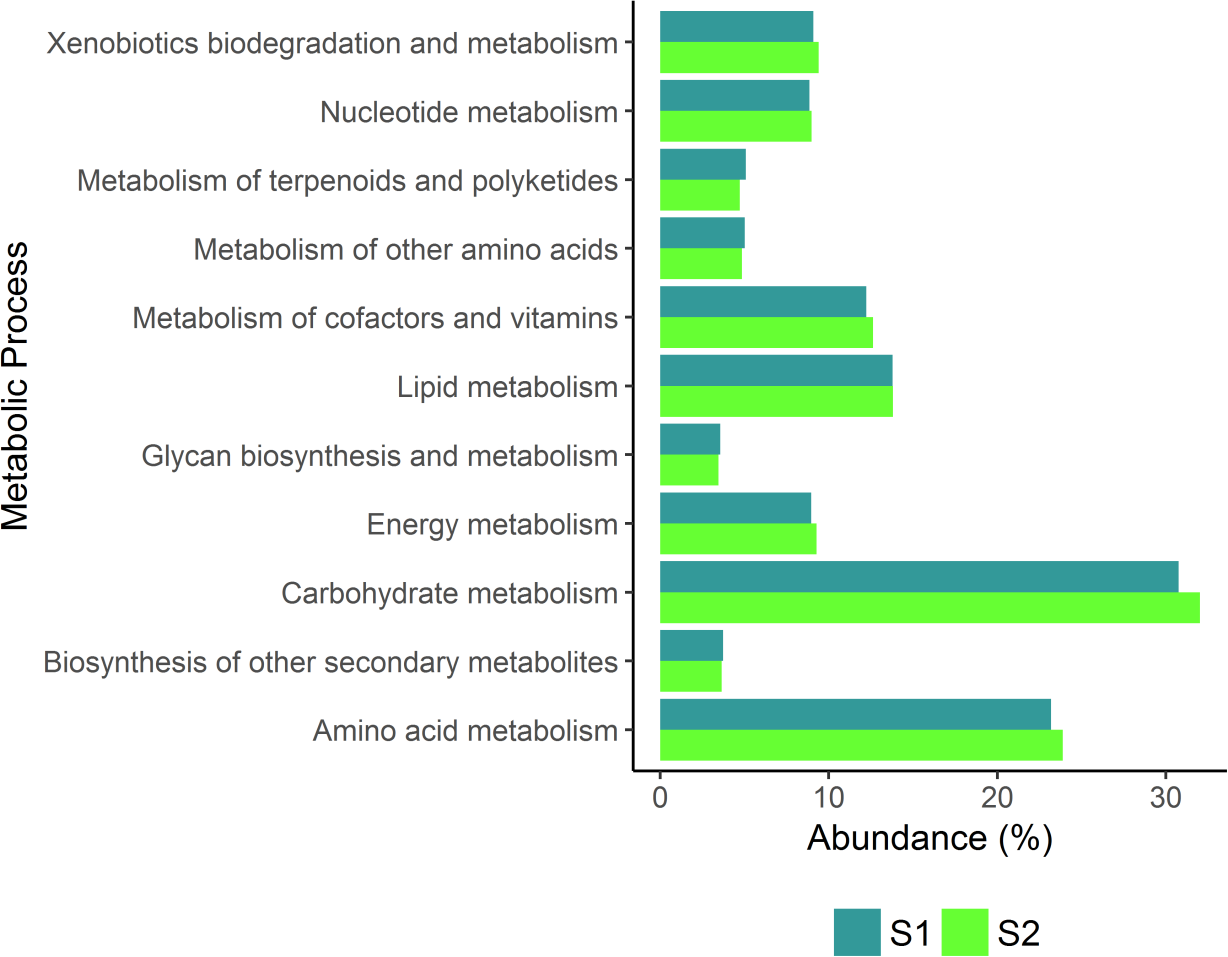
Comparison between the relative abundance of the metabolic processes performed in the non-intervened ecosystem (S1) and in the ecosystem intervened by agricultural processes (S2).

Metabolic processes were characterized according to the richness (i.e., the number of metabolic processes) and uniformity (i.e. the relative abundance of a particular metabolic process in a sample) [31]. Most of the metabolic diversity and the relative abundance of the metabolic processes, i.e., the ratio between the abundance of each metabolic process and the total numbers of all metabolic activities of each sample (expressed as a percentage), was maintained in both ecosystems. In both communities, the reactions were distributed in 140 metabolic modules, belonging to 11 functional metabolic pathways. **Fig 2**shows the relative abundance of the metabolic pathways in both ecosystems. Over 30% of the mapped reactions in the microbial communities were associated with carbohydrate metabolism, followed by amino acid (≈ 23%) and lipid metabolism (≈ 14%) in both ecosystems. Although it is possible that the loss in metabolic capabilities is present due to the lower amount of sequences obtained in S2, the similarity of the metabolic modules and the relative abundance in both ecosystems could indicate that the low number of enzymes on S2 is related to the agricultural intervention.

### The agricultural intervention of soils affects the diversity of microorganisms responsible for the regulation of biogeochemical cycles

The taxonomic profile obtained by MG-RAST showed that Bacteria are the dominant kingdom in both ecosystems studied, followed by the Eukarya, Archaea, and Viruses (**S9 Table**). The presence of Eukarya in both ecosystems suggests the need to consider the subcellular compartments into the modeling of the metabolic networks, since the Eukarya kingdom has subcellular compartments associated to specific organelles.

Using the microbial community diversity, we determined the relation between microbial soil composition and the regulation of biogeochemical cycles in the ecosystems. We evaluated the biogeochemical processes of carbon (CO_2_ fixation and respiration], nitrogen [nitrification, denitrification, and N_2_ fixation), and sulfur cycling (sulfate reduction and sulfur oxidation) [32]. As Bacteria and Archaea contain most of the microorganisms responsible for the regulation of the biogeochemical cycles, we focused on prokaryotes.

In the nitrogen cycle, over 26 genera, 11 families, and 3 orders of nitrogen fixing microbes were reported in the literature [33]. A total of 24 genera were found in both ecosystems. Overall, the non-intervened and agriculturally intervened soil samples, showed a similar percentage of genera implicated in nitrogen fixation. However, the non-intervened soil sample showed a higher abundance of diazotrophic microorganisms (**S10 Table**).

Nitrifying bacteria carry out the process of nitrification, the biological oxidation of ammonia (NH_4_^+^) to nitrite (NO_2_^-^), followed by the oxidation of nitrite to nitrate (NO_3_^-^). Bacterial genera known to be ammonium and nitrite oxidizers such as *Nitrosomonas*, *Nitrococcus*, *Nitrobacter*, and *Nitrospira* were identified in both ecosystems studied.

In the denitrification process, nitrates get reduced into inert gaseous nitrogen [N_2_]. This process could be performed in both microbial communities by bacteria belonging to the genus *Pseudomonas* and *Clostridium*. For all steps of the nitrogen cycle analyzed, we found that the percentage of microorganisms was higher for the non-intervened ecosystem. However, the intervened ecosystem showed higher abundances in the genera *Nitrospira* and *Nitrosomonas*, which are involved in the nitrification process.

The sulfur cycle is fundamental as it provides an element that is constituent of cofactors and proteins and thus, is essential for life. The sulfur atom has the capacity to occur in various oxidation states ranging from -2 (sulfide) to +6 (sulfate). Most of the sulfur redox reactions are not spontaneous and are catalyzed by prokaryotes capable of using several inorganic sulfur compounds in their metabolic processes [34]. For both soil samples studied, several sulfate-reducing bacteria were found. Among these, were *Desulfobacterales*, *Desulfovibrionales*, *Syntrophobacterales* (*Deltaproteobacteria*), and the genus *Desulfotomaculum* belonging to the *Firmicutes* phylum. Additionally, two genera of Archaea, *Thermocladium* and *Caldivirga*, capable of reducing sulfate, were also found. Both analyzed soil samples showed the same profile distribution of sulfate reducing microorganism (**S11 Table**).

Oxidation of sulfur to sulfate is one of the major processes in the sulfur cycle. In both soil ecosystems we found several genera of sulfur oxidizing Bacteria and Archaea. The overall profile of abundances of microorganisms for this process did not show a tendency bias towards any of the samples analyzed. A representative proportion of contigs (382) affiliated to the phylum *Crenarchaeota*, class *Thermoprotei*, which are thermophilic and sulfur-dependent organisms, was found in both soil samples. However, the soil sample collected in the agriculturally intervened ecosystem showed a 6% increase in the number of sequences associated to this phylum.

For carbon cycling we found the presence of *Cyanobacteria* in both metagenomic datasets. This phylum of bacteria obtain their energy through photosynthesis and fix CO_2_ using solar energy mainly by the reductive pentose phosphate cycle [35]. The photosynthesis process identified in the non-intervened sample coincided with the higher abundance of *Cyanobacteria* such as *Gloeobacter* and *Cyanobium*, while the intervened soil sample showed higher abundance of *Cyanothece* and *Synechococcus* genera. Furthermore, both soil samples contained autotrophic Bacteria and Archaea with additional carbon fixation pathways. One of these additional pathways is the reductive citric acid cycle found in the phylum *Chlorobi*, composed of aerobic and micro-aerobic bacteria. The taxonomic analysis showed the presence of the non-sulfur bacterial family *Chloroflexaceae*, which carry out the 3-hydroxypropionate bi-cycle to fix carbon. The last additional pathway for carbon fixation is the hydroxypropionate-hydroxybutyrate cycle, found in aerobic Archaea such as *Crenarchaeota* and *Metallosphaer* (**S12 Table**). In the alternative carbon fixation pathways, both soil samples displayed a similar abundance distribution of microorganisms. However, the intervened soil sample showed greater abundances of Archaeal genera. In both soil samples analyzed most of the Archaeal taxa corresponded to methanogens within the *Euryarchaeota* phylum. These represented 82% and 69% of Archaea in the non-intervened soil and in the intervened soil, respectively.

### The metabolic network compartmentalization allows the integration of the taxonomic profile and the functional characterization of the microbial communities

**Fig 3:**
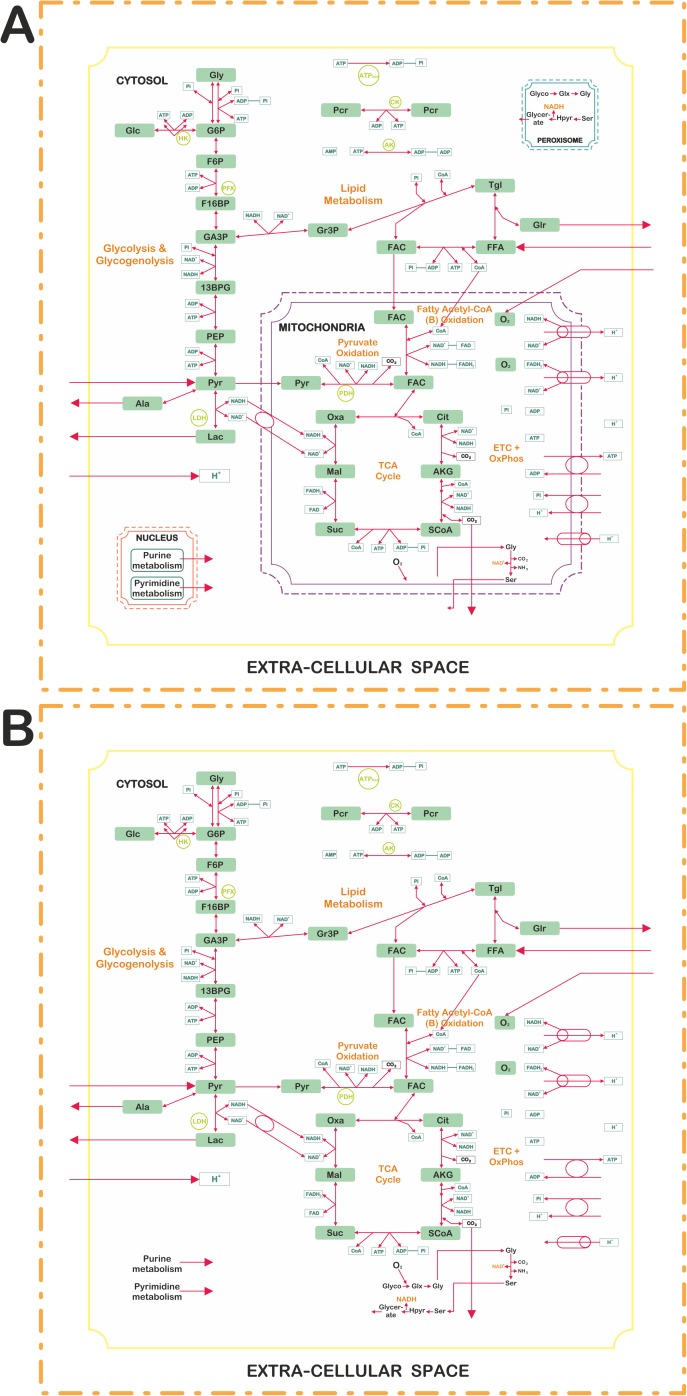
Schematic representation of compartmentalization. **A.** Compartmentalized metabolic network representation with four compartments evaluated. **B.** Non-compartmentalized metabolic network representation.

Understanding the metabolic interactions of microbial ecosystems is challenging given that the cellular localization of metabolic functions among the members of the ecosystem is often unknown. Therefore, it is important to investigate the effects of compartmentalization of metabolites and reactions over the flux balance predictions, and the topological features and connections between metabolic pathways in models of the microbial communities. In **Fig 3**the cellular compartments taken into account were schematized and compared against the non-compartmentalized model.

Using our metagenomic annotation we implemented the CELLO algorithm [23] to obtain preliminary localization of reactions for both datasets. Reactions of the non-intervened soil sample were localized into nine cellular compartments, while reactions of the agriculturally-intervened soil sample were localized into seven compartments (**S13 Table**). The reactions associated with the vacuole and the lysosome compartments were absent in the soil sample intervened by agricultural process and were almost negligible for the non-intervened soil sample [seven reactions were associated with the vacuole and 20 reactions were associated with the lysosome]. For this reason, we discarded the presence of these two compartments within both metabolic reconstructions.

To obtain more refined compartmentalized models, we used the taxonomic profiles to identify the subcellular localization of reactions in specific microorganisms. The species taken into account were: *Saccharomyces cerevisiae*, *Arabidopsis thaliana*, *Chlamydomonas reinhardtii*, *Cyanothece* sp. ATCC 51142, *Synechocystis sp*., *Leishmania major*, and *Pseudomonas putida*. The metabolic network reconstruction of these species are reported in the MetanetX database [20]. Only these metabolic network reconstructions were considered given that they are the unique compartmentalized models included in our taxonomic profiles and are reported in MetaNetX database.

The resulting consensus networks contained five compartments: cytoplasm, mitochondrion, nucleus, peroxisome, and the extracellular space. Due to the abundance of prokaryotic microorganisms the compartment with greater number of metabolites was the cytoplasm, followed by the metabolites related to the mitochondria, which is the organelle that carries out important energy processes (**Table 2**).

**Table 2:** Number of metabolites associated to each one of the compartments in the initial reconstructions. Importantly, a metabolite may be present in more than one compartment. This information excludes generic metabolites (metabolites that do not have specific stoichiometric coefficient: RNA, DNA, generic lipids and glycan).

Compartments	S1	S2
**Cytoplasm**	1928	1851
**Mitochondrion**	569	566
**Nucleus**	276	196
**Peroxisome**	74	60
**Extracellular space**	295	295

S1: Non-intervened soil sample. S2: Intervened soil sample.

A total of 740 transport reactions were included initially in both models, of which 571 were involved in transporting metabolites between the cytoplasm and the extracellular space and 168 reactions involved in the transport of metabolites between the cytoplasm and the other cellular compartments.

The initial compartmentalized network of the non-intervened soil sample S1 (S1_C) included 3,440 reactions and 3,142 distinct chemical compounds. We differentiated compounds localized in a different cellular compartment (e.g., NAD present in the cytoplasm, mitochondrion, peroxisome, and nucleus is classified as four different compounds). A total of 45% of the reactions were considered reversible, 33% were irreversible, and 22% were involved in transport processes. On the other hand, the initial compartmentalized metabolic reconstruction of the intervened soil sample S2 (S2_C) resulted in a final network of 3,235 reactions and 2,968 distinct metabolites. In this model, 47% of the reactions were considered reversible, 30% were irreversible, and 23% were transport reactions.

To address the effect of removing cellular compartments on the model’s capability of prediction, we created an additional non-compartmentalized network for each metagenome. In these models we considered only two compartments: cytoplasm (where all the metabolic processes occur) and extracellular space, to ensure the exchange of metabolites between microbial communities and the environment. The non-compartmentalized model of the non-intervened soil (S1_NC) had 2,237 metabolites and 2,603 reactions, while the non-compartmentalized model of intervened sample (S2_NC) had 2,153 chemical compounds and 2,497 reactions. A total of 571 transport reactions were involved in the transportation of metabolites between the cytoplasm and the extracellular space in both samples.

### Metabolic network curation is a critical step to restore the metabolic fluxes within the networks

The first reconstruction step led to a set of reactions that were used as the basis for the construction of a stoichiometric representation of each metabolic model. This representation permitted identifying the gaps in the networks, the calculation of the mass balances around each metabolite, and the identification of the no-production and no-consumption metabolites. A total of 1,295 and 1,211 metabolites for the non-intervened compartmentalized network (S1_C) and for the intervened compartmentalized network (S2_C), respectively, could not be consumed by any of the reactions in the network.

**Fig 4:**
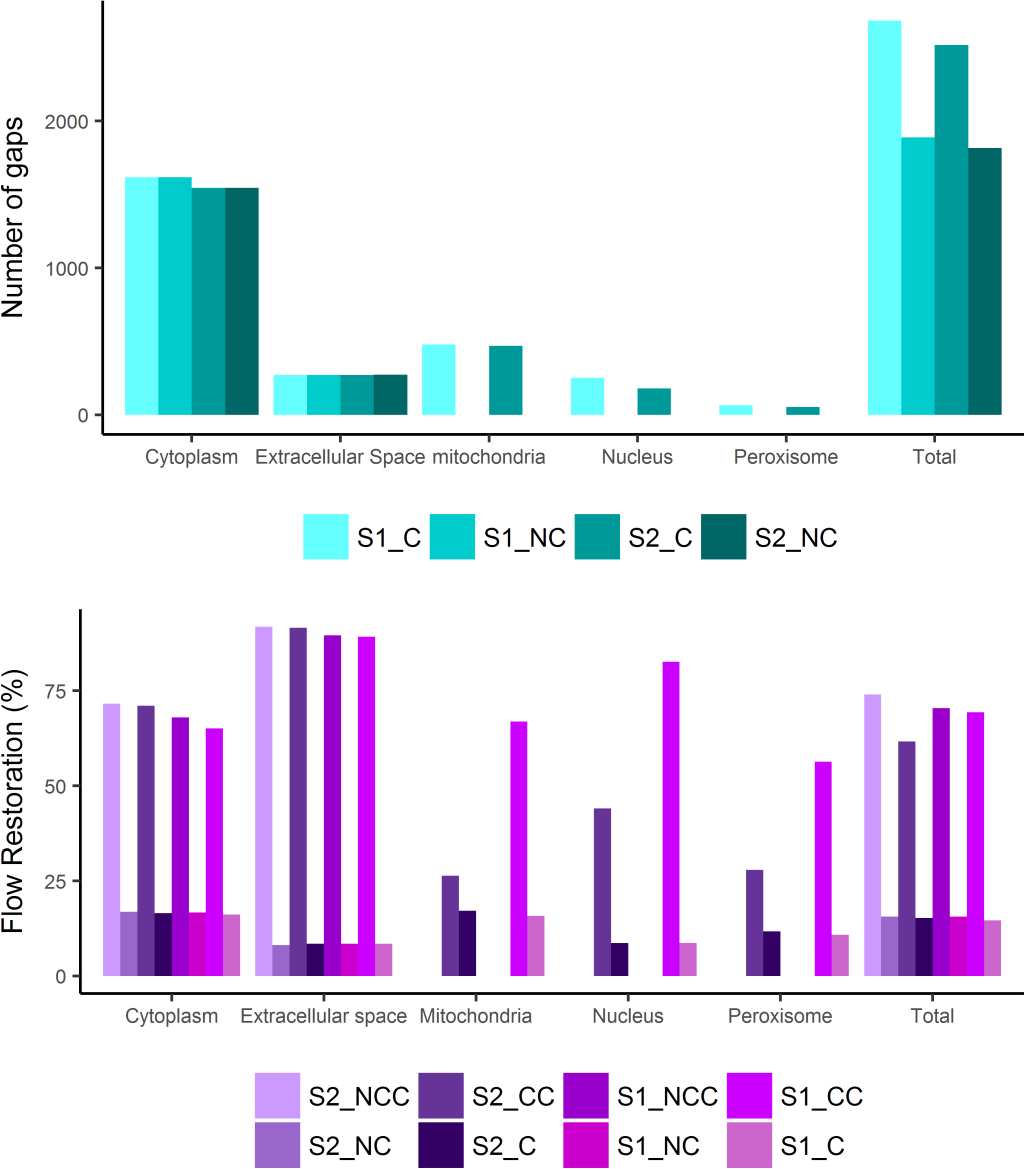
Results of the compartmentalized metabolic network curation. **A.** Total metabolite problems found in each one of the compartments evaluated. **B.** Percentage of reconnection before and after the Gapfilling process. S1_C: compartmentalized, non-intervened soil sample. S2_C: compartmentalized, intervened soil sample. S1_NC: non-compartmentalized, non-intervened sample. S2_NC: non-compartmentalized, intervened sample. S1_CC: compartmentalized and curated, non-intervened sample. S2_CC: compartmentalized and curated, intervened sample. S1_NCC: non-compartmentalized and curated non-intervened soil sample. S2_NCC: non-compartmentalized and curated intervened soil sample.

A total of 1,389 and 1,305 metabolites that could not be produced under any uptake condition were identified for samples S1_C (non-intervened) and S2_C (intervened), respectively. As shown in **Fig 4A**, most of the gaps for both metagenomes are in the cytoplasm, followed by the mitochondria and extracellular space. The percentage of disconnected metabolites in each compartment is similar for both soil samples [**Fig 4B**]. In both microbial communities a considerable number of biochemical reactions and metabolic functions may be missing. Results revealed that none of the cellular compartments contain completely connected metabolites (**S14 Table**).

The GapFill algorithm [24] was used to solve different gaps following three main steps. First, the gaps found in the cytoplasm were filled; second, exchange reactions between the cytoplasm and the other compartments were identified and added; and finally, the gaps in the remaining compartments were completed and curated separately. As shown in **Table 3**, there was a high number of common gaps between the cytoplasm and the other cellular compartments. Notably, around 50% of the gaps of the mitochondria and between 9 to 40% of the gaps in the inner compartments are shared with the cytoplasm. Therefore, the gaps in the inner compartments can be solved identifying the production pathways of cytoplasm and adding adequate exchange reactions.

**Table 3:** Number of common problem metabolites between the cytoplasm and the inner compartments.

	S1	S2
**Cytoplasm Mitochondrion**	247	217
**Cytoplasm Nucleus**	49	28
**Cytoplasm Peroxisome**	6	6
**Cytoplasm Extracellular space**	108	86

S1: Non-intervened soil sample; S2: Intervened soil sample.

A total of 1,459 and 1,203 unique reactions of MetanetX database [20] were added to the initial non-intervened compartmentalized S1_C and intervened compartmentalized S2_C models, respectively. Additionally, 94 exchange reactions were added to enable the production and consumption of the metabolites showing gaps. **Table 4**shows the total number of metabolites and reactions added to each compartment. As expected, it was necessary to add more reactions and metabolites to restore the non-intervened compartmentalized network (S1_C), since this model had a higher number of reactions and metabolites; it was more difficult to restore the connectivity of the network. Added reactions were manually evaluated in order to ensure that they were present in previous identified taxa.

**Table 4:** Metabolic characterization of the consensus metabolic networks.

	S1_C	S2_C	S1_NC	S2_NC
**Total reactions**	5,607	4,557	4,060	3,693
**Added reactions**	2,073	1,322	1,939	1,677
**Total transport reactions**	834	834	576	569
**Total metabolites**	3,838	3,376	2,664	2,429
**Number of metabolites by compartment**				
**Cytoplasm**	2,222	2,110	2,368	2,134
**Mitochondria**	783	580	NA	NA
**Nucleus**	518	311	NA	NA
**Peroxisome**	119	79	NA	NA
**Extracellular space**	296	296	296	296

S1_C: non-intervened soil sample, compartmentalized. S2_C: intervened soil sample, compartmentalized. S1_NC: non-intervened soil sample, non-compartmentalized. S2_NC: intervened soil sample, non-compartmentalized.

The non-intervened soil sample reached a higher number of connected metabolites, compared with the achieved reconnection in the inner compartments of the intervened soil sample. In the non-intervened soil sample (S1_C) we reached the higher percentage of metabolic curation, in the extracellular space (89.19%), followed by the nucleus (82.63%), the mitochondria (66.92%), the cytoplasm (65.08%) and the peroxisome (56.30%). On the other hand, for the agriculturally-intervened soil (S2_C) the extracellular space also reached a higher percentage of metabolic curation (91.55%), followed by the cytoplasm (71.04%), the nucleus (44.05%), the mitochondria (26.38%) and the peroxisome (27.84%) (**Fig 4B** and **S15 Table)**. The lower percentages of metabolite connections within inner compartments in the agriculturally-intervened soil may be explained by at least two reasons: i) that the used database does not contain the amount of reactions capable of restoring the flow in the inner compartments for the intervened soil sample; ii) that there was not enough information in the curated process for the exchange reactions between the inner compartments and the cytoplasm for this sample. Consensus networks for both soil samples are shown in **Table 4.**

We analyzed the effects of the compartmentalization process in the number of gaps and in the resulting network after the curation process. For the non-compartmentalized network of the non-intervened soil (S1_NC), 993 no-production metabolites and 895 no-consumption metabolites, were identified. Meanwhile, for the non-compartmentalized network of the intervened soil (S2_NC), 957 no-production metabolites and 859 no consumption metabolites were identified. As shown in **Fig 4B**, around 16.5% and 8% of the connected components in the cytoplasm and in the extracellular space, respectively, were obtained by the non-compartmentalized models. The result of the non-compartmentalized models showed less non-connected metabolites compared to the compartmentalized models.

### The flux balance analysis showed that fluxes associated with the nitrogen cycle affect the regulation of the carbon and sulfur cycles

The FBA employs a linear programming (LP) strategy to obtain a metabolic flux distribution that is optimized towards a specific cellular objective. This analysis is subject to a set of physicochemical and thermodynamic constraints [36]. The flow predictions of FBA are dependent on the objective function selected for the analysis. The most common objective function involves the maximization of biomass synthesis. However, the rate of biomass growth is not necessarily the objective of the microbial ecosystems, because the biological objective of these communities will probably be to optimize exploitation of nutrients and response to perturbations. Furthermore, based on previous findings by our research group, the objective function in this type of ecosystem was associated to biogeochemical cycles because of the high content of minerals in the environment [6]. We evaluated different objective functions associated with the optimization of nitrogen, carbon, and sulfur cycles and analyzed metabolic pathways such as the tricarboxylic acid cycle (TCA) and fatty acid elongation in the mitochondria (**Table 5**). Through these objective functions we optimized each model and their dependency with the presence of cellular compartments.

**Table 5:** Objective functions for the carbon, sulfur, and nitrogen cycles.

**NITROGEN CYCLE**
8 H(+) + 8 reduced ferredoxin + 16 H_2_O + 16 ATP + 1 dinitrogen < = > 2 NH_4_(+) + 1 dihydrogen + 8A + 16 ADP + 16 phosphate
1 ubiquinone + 1 H_2_O + 1 hydroxylamine < = > 1 NH_4_(+) + 1 O_2_ + 1 Ubiquinol
5 H(+) + 1 nitrite + 2 ferrocytochrome c = 2 ferricytochrome c + 1 H_2_O + 1 hydroxylamine
2 H(+) + 2 ferrocytochrome c + 1 dinitrogen oxide < = > 2 ferricytochrome c + 1 H_2_O + 1 dinitrogen
**SULFUR CYCLE**
3 H_2_O + 3 NADP(+) + 1 hydrogen sulfide < = > 1 H(+) + 1 sulfite + 3 NADPH
3 H_2_O + 3 A + 1 hydrogen sulfide = 8 H(+) + 1 sulfite + 3 reduced ferredoxin
2 H(+) + 2 ferrocytochrome c + 1 sulfate < = > 1 sulfite + 2 ferricytochrome c + 1 H_2_O
1 hydrogen peroxide + 1 sulfate < = > 1 sulfite + 1 H_2_O + 1 O_2_
**AMMONIA CYCLE**
1 acetyl phosphate[2–] + 1 ADP < = > 1 acetate + 1 ATP
Oxaloacetate[c] < = > Oxaloacetate[m]
Acetyl-CoA[c] < = > Acetyl-CoA[m]
Oxoglutarate[c] < = > Oxoglutarate[m]
Citrate + CoA < = > Acetyl-CoA + H_2_O + Oxaloacetate
ATP + Oxaloacetate < = > ADP + Phosphoenolpyruvate + CO2
Acetyl-CoA + Enzyme N6-(dihydrolipoyl)lysine < = > CoA + (Dihydrolipoyllysine-residue acetyltransferase) S-acetyldihydrolipoyllysine
**FATTY ACID ELONGATION**
ATP + Hexadecanoic acid + CoA < = > AMP + Palmitoyl-CoA + Diphosphate
Acyl-CoA + Acetyl-CoA < = > CoA + 3-Oxoacyl-CoA
acetyl-CoA[c]< = = >acetyl-CoA[m]

Since most of the microorganisms responsible for the regulation of the biogeochemical cycles are prokaryotes, we focused on the compartment associated with the cytoplasm to carry out a comparative study of the biogeochemical features of the ecosystems. We evaluated the percentage of active flows reached with each analyzed objective function, i.e., we determined the number of flows different from zero, when optimizing a specific objective function.

In the case of the nitrogen cycle, 52.92% of the overall metabolism and 44.37% of the reactions of the cytoplasm were activated for the non-intervened compartmentalized soil sample (S1_C). For the intervened compartmentalized soil sample S2_C, activation was obtained for 45.86% of the global metabolism and 60.75% of the flow in the cytoplasm. The presence of reactions involved in the cycling of sulfur and carbon is remarkable when the models maximize the nitrogen cycle. These flow parameters reflect the interdependence of all biogeochemical cycles in the ecosystems. A greater number of active fluxes related with the carbon and sulfur cycles in the non-intervened compartmentalized soil sample (S1_C) were found, particularly fluxes associated with the sulfur cycle. In the sample S1_C 33.33% of the fluxes within the sulfur cycle remained active, while in the intervened compartmentalized soil sample (S2_C) only 15.56% of the fluxes were activated, when the model maximized the nitrogen cycle.

The behaviors of the three steps considered for the nitrogen cycle (i.e. nitrogen fixation, nitrification, and denitrification were evaluated. In the intervened and non-intervened models, 100% of the fluxes related with the nitrogen fixation and nitrification processes were active. In the non-intervened compartmentalized soil sample (S1_C) 44.44% of the fluxes were related to the denitrification process, but only 22.2% of the fluxes were active in the intervened compartmentalized soil sample (S2_C) (**Table 6**).

**Table 6:** Effect of biogeochemical objective functions on other cycles. S1_C: non-intervened soil sample, compartmentalized.

	S1_C [%]	S2_C [%]	S1_NC [%]	S2_NC [%]
**NITROGEN OBJECTIVE FUNCTION**				
Nitrogen cycle	35.90	33.33	28.21	30.77
Carbon cycle	30.95	23.81	15.48	22.62
Sulfur cycle	33.33	15.56	28.89	22.22
Nitrogen fixation	100	100	100	100
Nitrification	100	100	100	100
Denitrification	44.44	22.22	55.56	33.33
**SULFUR OBJECTIVE FUNCTION**				
Nitrogen cycle	28.21	33.33	20.51	15.38
Carbon cycle	27.38	26.19	21.43	22.62
Sulfur cycle	42.22	20.00	28.89	20.00
Assimilatory sulfate reduction	60	40	60	20
Dissimilatory sulfate reduction	100	66.67	100	33.33
Thiosulfate oxidation (thiosulfate-dehydrogenase-rxn]	100	100	100	100
**CARBON OBJECTIVE FUNCTION**				
Nitrogen cycle	20.51	17.95	15.38	15.38
Carbon cycle	28.57	27.38	21.43	22.62
Sulfur cycle	40.00	13.33	22.22	17.78

S2_C: intervened soil sample, compartmentalized. S1_NC: non-intervened soil sample, non-compartmentalized. S2_NC: intervened soil sample, non-compartmentalized.

When the target of the metabolic networks was the optimization of the sulfur cycle, the non-intervened compartmentalized soil sample (S1_C) reached a total of 53.15% of the overall reactions and an activation of 44.55% of the cytoplasm’s fluxes. On the other hand, the intervened compartmentalized soil sample (S2_C) showed less activation of the global fluxes (46.50%), and higher active fluxes associated to the cytoplasm (62.132%). Due to the interdependent relation between all biogeochemical cycles, the activation of a considerable number of fluxes linked to the cycle of the other evaluated chemical compounds was observed. For the sulfur cycle the fluxes related with sulfate reduction and sulfur oxidation were analyzed. Regarding sulfate reduction, assimilatory and dissimilatory pathways were considered. Higher percentages of coverage for both pathways were found for the non-intervened ecosystem (**Table 6**). The absence of fluxes associated with sulfur oxidation in both ecosystems is notable, despite the presence in the taxonomic profile of microorganisms able to perform this process.

Due to the complexity of the pathways associated with the carbon cycle, we focused on methane metabolism, carried out mainly by methanotrophs and methanogens in the global carbon cycle. When the microbial communities optimize this cycle, the non-intervened soil sample S1_C showed a higher number of active fluxes in the whole network, but less coverage of fluxes in the cytoplasm compared to soil sample S2_C (**Table 6**).

In the case of the objective functions associated to the cytoplasm, the effect of compartments in the optimization was not observed, nor was there a significant difference between the percentages of global coverage of the metabolic fluxes. In order to evaluate the effect of the presence of compartments in the models, with respect to the flux predictions and to the results of the optimization process, the objective functions associated to a specific inner compartment [such as the tricarboxylic acid cycle (TCA) and the pathway for fatty acid elongation, characteristic of the mitochondria) were evaluated. In both cases, the main reactions associated to each metabolic pathway as well as the exchange reactions required for the transport of metabolites between the cytoplasm and the mitochondria were considered in the objective function (**Table 5**).

The optimization of the TCA cycle showed the same values of the objective function for both ecosystems. These analyses demonstrate the effect of the addition of inner compartments into the model and the importance of considering existing reactions between the cytoplasm and the added compartments. The optimal values obtained were higher (3 mmol gDW^-1^ h^-1^) when the main TCA and exchange reactions were included, compared to the results when we did not consider the interchanges of metabolites between cytoplasm and mitochondria (2 mmol gDW^-1^ h^-1^).

To assess the importance of the inner compartments in the construction of the models, we evaluated the same objective function in the non-compartmentalized networks and obtained lower values for the optimal solution (1.5mmol gDW^-1^ h^-1^) with respect to the compartmentalized networks (**S16 Table).**

In the case of the objective functions associated to fatty acid elongation, the same interdependent relation between the presence of inner compartments and the exchange reactions, with the results of the optimization process was observed. Notably, the non-intervened soil sample clearly showed that when the transport of acetyl-CoA from the cytoplasm to mitochondria was not taken into account, the pathway for fatty acid elongation could not be activated, resulting in a value of objective function equal to zero.

### Compartmentalized metabolic networks at a metagenomics scale intervened and non-intervened by agricultural processes conserved the main topological characteristics of the non-compartmentalized metabolic networks at a genomics and metagenomics scale

**Fig 5:**
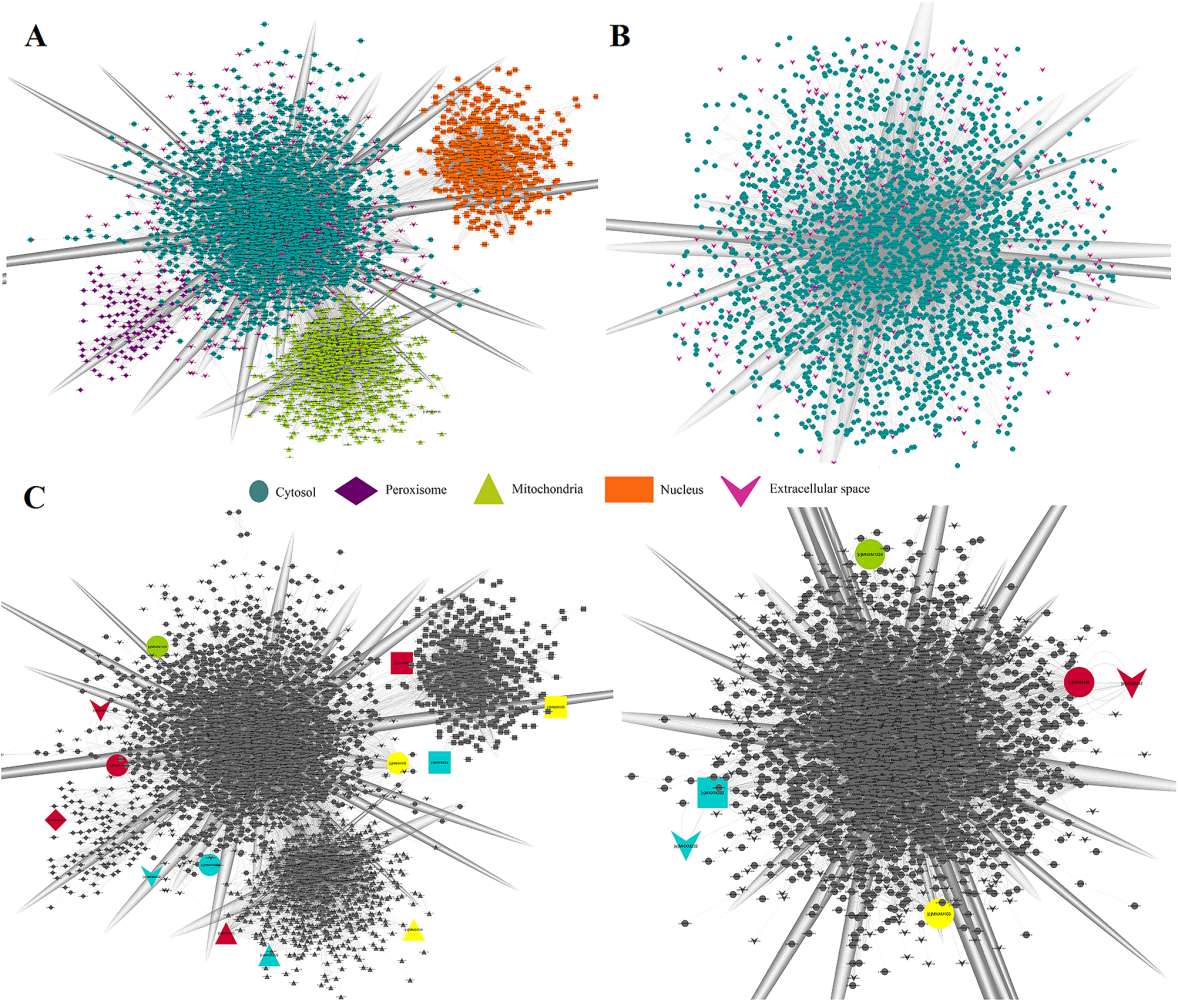
Graph representation of metabolic networks: (**A)** the compartmentalized, non-intervened soil sample and (**B)** the non-compartmentalized, non-intervened soil sample. In metabolic networks, the nodes represent the metabolites and the links represent the biochemical reactions. **(C)** Comparison between metabolites in compartmentalized and non-compartmentalized networks. The same color highlight the same metabolite in both networks.

The topological properties of models used to understand the architecture of metabolic networks was examined. In this way, the effect of agricultural intervention on soils and the presence of compartments on their structural features can be evaluated. As shown in **Fig 5**, a graphical representation of the metabolic networks was first established. In metabolic networks, the nodes represent the metabolites and the links represent the biochemical reactions. The models had a significant number of irreversible reactions; therefore, for each node it was possible to distinguish between incoming and outgoing links. Compartmentalized and non-compartmentalized networks contain the same set of unique metabolites. The differences between both networks are in terms of the metabolites localization more than type of metabolites (**Fig 5C**).

The main topological characteristic of the network is the degree distribution *P*(*k*), which shows the probability that a particular metabolite has exactly *k* reactions. The degree distribution *P*(*k*) allows us to distinguish between different types of biological networks. A power-law degree distribution for the compartmentalized and the non-compartmentalized networks was obtained. This distribution, characterizes scale free networks, in which the probability that a metabolite displays *k* reactions follows *P*(*k*) ≈ *k*^−*γ*^, where *γ* is the degree exponent that describes the role of the hubs in the system [37]. In directed networks the incoming and outgoing reactions (*k*) can be distinguished, therefore, a metabolite may participate as a reactant in *k* metabolic reactions following the power-law distribution *P*(*k*) ≈ *k*^−*γin*^ with *γ*_*in*_ ≈ 1.8, for compartmentalized and non-compartmentalized networks. For outgoing links a given metabolite may be produced by *k* different metabolic reactions following a similar distribution [37], *P*(*k*) ≈ *k*^−*γout*^ with *γ*_*out*_ ≈ 2 for compartmentalized and non-compartmentalized models. We obtained values of γ near or equal to 2, indicating that there are hierarchies of hubs, i.e. large numbers of metabolites with few connections, while highly connected metabolites are scarce.

A common feature of many metabolic networks is their small-world property, where all of the nodes in the networks are connected by a relatively short path to each other [38]. This property increases the network efficiency, minimizing the transition times between metabolic states [39]. This topology feature can be characterized by the average path length and by the diameter of the network, defined as the average and maximum number of links between all pairs of nodes, respectively [38]; in metabolic networks these links correspond to the biochemical reactions connecting two metabolites. In compartmentalized metabolic networks, greater values of diameter than the reported for others metabolic networks were found (**Table 7**]. The unique topological significant difference between both soil samples was the network diameter, higher for the non-intervened soil sample (S1), because this ecosystem has more metabolites and reactions, therefore more nodes and edges to connect. The average path length for all analyzed metabolic networks was ≈3.3.

**Table 7:** Global topological properties. S1_C: non-intervened soil sample, compartmentalized.

	S1_C	S2_C	S1_NC	S2_NC
**Clustering coefficient**	0.178	0.163	0.199	0.188
**Network diameter**	13	9	9	9
**Network radius**	1	1	1	1
**Characteristic path length**	3.718	3.549	3.057	3.087
**Avg. number of neighbors**	7.639	7.274	7.718	7.754
**Number of nodes**	3935	3373	2662	2427
***γ***_***in***_	1.880	1.790	1.703	1.729
***γ***_***out***_	2.090	1.921	1.880	1.913

S2_C: intervened soil sample, compartmentalized. S1_NC: non-intervened soil sample, non-compartmentalized. S2_NC: intervened soil sample, non-compartmentalized.

Another important topological feature of the networks is the average clustering coefficient, which is a measure of the potential modularity of the network. Contrary to what was expected, the average clustering coefficient for metagenomic networks was lower than the reported for the organisms studied by Jeong *et al*. [37] and for the metabolic network of *Escherichia coli* [40]. This might be due to the fact that metabolic networks at a metagenomics scale have an average clustering coefficient similar to that expected for scale-free networks, characterized for a large number of nodes with few connections and scarce highly connected nodes.

On the other hand, we ranked each metabolite in the networks according to their topological properties, to detect the central and intermediate nodes that determine the topology of the network (**S17, S18, S19 and S20 Tables**). As expected according to measures of centralities such as *in-out* degree and edge count, the central metabolites for all evaluated networks were H, H_2_O, ATP, NAD, NADH, NADP, CO_2_, Pi, NH_4_, and O_2_, which play a central role in the energetics and central pathways of cells. These results are in agreement with the PCA, which showed that these were the most central metabolites because they were connected with almost every other metabolite. Notably, in the compartmentalized models the most connected metabolites were distributed between the cytoplasm and the mitochondria, which also showed the higher values of intermediation, indicating that these compounds control the fluxes between the cytoplasm and the mitochondria.

## Discussion

Through a systems biology approach, we were able to construct a curated metabolic network that allowed us to determine the effect of modeling microbial communities as compartmentalized meta-organisms in contrast to more simple non-compartmentalized models. The proposed models highlight specific information about ecosystems that are generally overlooked in non-compartmentalized models, like the effect of transport reactions between inner compartments and the cytoplasm in the metabolic processes, and furthermore provide more accurate metabolic flow patterns. Based on these metabolic reconstructions we were able to determine the effect of agricultural intervention on the metabolic structure, microbial diversity, and the topological features of soil ecosystems in a Colombian Natural Park. We found that agricultural intervention of soils leads to a loss of metabolic capabilities of the microbial communities.

The abundance of metabolic processes that occurred in the non-intervened soil sample, showed a higher numbers of enzymes, reactions, and metabolites, than the agriculturally intervened one. Consequently, the non-intervened soil had higher capabilities to regulate ecological processes. In both non-intervened and intervened ecosystems over 30% of the reactions were associated with carbohydrate metabolism, followed by amino acid, and lipid metabolism. These results are in concordance with those found in a study that investigated the functional metagenomic profiling of nine biomes [31] and with the analysis of biogeochemical cycles in metagenomes in a Colombian Natural Park [6], indicating that the two analyzed ecosystems conserve the same metabolic distribution reported for biomes under different environmental conditions, regardless of the agricultural intervention processes or extreme environmental features. This may be explained by the fact that sequences related to these metabolic pathways are relatively stable and shared among most individuals that make up the microbial community [6].

Despite the differences regarding the number of enzymes, reactions, and metabolites, no significant differences were found between microbial communities in the non-intervened and agriculturally intervened soil samples. Thus, it was not possible to determine a distinguishing metabolic profile for each of the analyzed microbial communities. However, the FBA and the taxonomic profiles, provided information about the main species that were influencing the biogeochemical cycles on each of the ecosystems. The non-intervened ecosystem had a higher abundance of microorganisms responsible for the regulation of biogeochemical cycles. Furthermore, the FBA showed that the agriculturally-intervened soil had fewer active metabolic flows associated with the regulation of biogeochemical processes. The values of the metabolic fluxes obtained cannot represent the studied ecosystem properties, but the metabolic pathways related with these fluxes showed the effect of modifying the objective function on the FBA result. In concordance with other metabolic studies of microbial ecosystems [6], the flow parameters obtained reflects an interdependence of all biogeochemical cycles in the ecosystems, since we found the strong presence of all biogeochemical metabolism fluxes in all objective functions evaluated. The nitrogen cycle plays a key role in the microbial communities of both agriculturally non-intervened and intervened soils. Remarkably, maximization of the nitrogen cycle significantly contributes to the total metabolic flux of the carbon and sulfur cycles and displays an important contribution in the activation of the whole metabolic network. These results were supported by the taxonomic analysis that showed that the most important differences between the non-intervened and agriculturally-intervened soil samples were related to the abundance of nitrifying bacteria, attributed to the need of transforming greater amounts of ammonia, which result from the use of fertilizers in the intervened ecosystem.

Through the FBA we were able to analyze the effect of modeling the microbial ecosystem as a compartmentalized meta-organism. The flow parameters associated to a specific compartment showed the importance of considering transport reactions between inner compartments and the cytoplasm, because these reactions have an important effect in the final results of the metabolic fluxes obtained. The results of the FBA for the analyzed objective functions showed an important dependency between the number of active fluxes on each compartment and the percentage of flux reconnections; this effect can be noted in the intervened compartmentalized soil sample (S2_C), where less restoration of flows in the inner cellular compartments were achieved, and therefore, a lower number of associated fluxes.

The topology of the reconstructed metabolic networks followed the power-law of node degree distribution. This feature of true complex biological networks was previously reported by our research group in studies of the topology of metabolic networks at a metagenomics scale [6]. The topological analysis showed that the average path length for all analyzed metabolic networks (≈ 3.3) was the same than that reported for the 43 organisms studied by Jeong *et al*. [37]. The fact that the networks at a metagenomics scale have the same average path length than the metabolic networks of individual organisms, is possible only if with increasing network complexity, metabolites are increasingly connected to maintain relatively constant the average path length of the metagenomic network [37]. The average clustering coefficient obtained for the metagenomics networks was lower than that reported for the organisms studied by Jeong *et al*. [37], as well as for the metabolic network reported for *E*. *coli* [40]. Metabolic studies at a genomics scale report high clustering coefficients, suggesting a modular organization of the networks. In these metabolic networks the nodes have approximately the same number of links, in contrast with the features of scale-free metabolic network [41]. Meanwhile, the metabolic networks at a metagenomics scale, showed an average clustering coefficient similar to those expected for scale-free networks, where there is a large number of nodes with few connections while highly connected nodes are scarce [42]. The topological analysis allowed us to infer two important characteristics of the evaluated networks: i) There are no significant differences between the structures of the networks of the intervened and non-intervened soil samples, or between compartmentalized or non-compartmentalized networks. ii) Metagenomic scale metabolic networks conserve the main characteristics of the metabolic networks at the genomic scale. The similarity between both ecosystems might reflect limitations in knowledge of the environments and the large amounts of un-annotated sequences present in their metagenomes.

To address the impact of the metagenomic scale on the metabolic reconstruction process, we compared the obtained results with those reported for the genome-scale networks of the non-compartmentalized model of *E*. *coli* and the multi-compartment model of *S*. *cerevisiae*. Significant differences between the percentages of disconnected metabolites in the genomic-scale and metagenomic-scale models were found. Kumar et al. [24] showed that about 10.4% of all metabolites in the *E*. *coli* model were disjointed from the rest of the metabolism, while in the non-compartmentalized models of the microbial communities about 84.4% of all metabolites were disconnected. In the case of the multi-compartment model of *S*. *cerevisiae*, Kumar et al. [24] reported that approximately 30% of all metabolites in the model were disjointed, in contrast with 85.1% of all metabolites disconnected in compartmentalized models of microbial communities. The presence of unbalanced metabolites in the metagenomics models with respect to the genomic models is not surprising due to the fact that the main gaps were identified in secondary metabolism pathways, which are more abundant in microbial communities than in individual microorganisms. Furthermore, *E*. *coli* and *S*. *cerevisiae* are extensively studied and well characterized models.

The effects of the process of compartmentalization on the gaps features of the network connection and in the resulting network after the curation process were assessed. The non-intervened soil sample contained more gaps, possibly due to the fact that this non-intervened ecosystem has more reactions and metabolites to reconnect. During the curation process, a higher percentage of reconnections for the non-compartmentalized models was obtained. This was because non-compartmentalized models did not show problems in the restoration of flows between inner compartments and the cytoplasm. These results show that the compartmentalization processes have a significant impact on the curation process. The reconstruction of compartmentalized metabolic networks for the microbial genomes is significantly more challenging than that for individual microorganisms because of the larger size of the networks and the considerable variation of metabolic activity between microorganisms [43]

## Conclusions

The modeling of microbial communities through this systems biology approach highlighted the main features of the studied ecosystems and the main and more determinant steps in the metabolic network reconstruction at a metagenomics-scale. One of the most critical steps in this study was the curation of the metabolic networks. This step had a significant effect on the results obtained for each model, because the flow restoration affected the interconnection between the central and the secondary metabolism, as well as the ability to interchange metabolites between the inner cellular compartments and the cytoplasm. Another important step in this metabolic reconstruction was the compartmentalization process given that a detailed distribution and cellular localization of metabolic functions at a metagenomic scale is very difficult to determine. This is because of the size of the networks and the considerable variation in the subcellular compartmentalization of the metabolic processes between different microorganisms. Therefore, the predicted cellular compartments for the metabolic networks may be improved by using other methods that complement the predictions of CELLO and the literature review.

The integration of the results of the taxonomic and metabolic profiles of the ecosystems and of the FBA, indicates that the non-intervened soil had a greater capacity to regulate biogeochemical cycles. On the other hand, the topological analysis showed that there were no significant structural differences between the compartmentalized and non-compartmentalized networks in either the intervened and non-intervened soil samples. Results also show that the metabolic networks at a metagenomics scale conserved the main characteristics of the metabolic networks at a genomics scale. At a metagenomics scale, metabolic networks fit to a type of scale-free network and conserve the small-world property, minimizing transition times between metabolic states, thus increasing the network efficiency.

The compartmentalized metabolic networks highlight information of ecosystems that is generally underestimated in non-compartmentalized models, like the role of metabolic fluxes involved in the transport between the inner compartments and the cytoplasm and between the cytoplasm and the extracellular space. Notably, the curation and reconstruction processes of compartmentalized networks are more challenging and more expensive in terms of time and computational resources. Therefore, it must be determined if the purpose of the research justifies the resources invested in the process of compartmentalization.

## Supporting information

S1 Table Metabolic genes identified and classified based on the enzyme commission for the non-intervened soil sample (S1). (XLSX)

S2 Table Metabolic genes identified and classified based on the enzyme commission for the intervened soil sample (S2). (XLSX)

S3 Table Mass, charge, and formula for each of the metabolites that made up a reaction in the non-intervened soil sample (S1). A pH of 7.2 was assumed.
(XLSX)

S4 Table Mass, charge, and formula for each of the metabolites that made up a reaction in the agriculturally intervened soil sample (S2). A pH of 7.2 was assumed.
(XLSX)

S5 Table MetanetX ID, Kegg ID, equation, equation description, standard Gibbs free energy, reaction balance, enzyme commission number (EC) and source of the reactions that made up the metabolic network in the non-intervened soil sample (S1). (XLSX)

S6 Table MetanetX ID, Kegg ID, equation, equation description, standard Gibbs free energy, reaction balance, enzyme commission number (EC) and source of the reactions that made up the metabolic network in the agriculturally intervened soil sample (S2). (XLSX)

S7 Table Analyses of the compartmentalized reconstructions reported in MetaNetX for organisms considered in the taxonomic profile obtained in this study. (XLSX)

S8 Table Transport and exchange reactions added to the models. The set of reactions added were those entirely composed by compounds belonging to the metabolic networks analyzed. Transport and exchange reactions were searched for in the BiGG and Metacyc databases.
(XLSX)

S9 Table Taxonomic profile obtained by MG-RAST. (XLSX)

S10 Table Taxonomic profile of the nitrogen cycle. (XLSX)

S11 Table Taxonomic profile of the sulfur cycle. (XLSX)

S12 Table Taxonomic profile of the carbon cycle. (XLSX)

S13 Table Initial compartments found by CELLO. (XLSX)

S14 Table Number of connected metabolites (presented as a percentage) at the beginning of each network: Non-intervened compartmentalized network (S1_C), non-intervened non- compartmentalized network (S1_NC), agriculturally-intervened compartmentalized network (S2_C), agriculturally-intervened non-compartmentalized network (S2_NC). (XLSX)

S15 Table Number of connected metabolites (presented as a percentage) after the restoration process for each network: Non-intervened compartmentalized network (S1_C), non-intervened non- compartmentalized network (S1_NC), agriculturally-intervened compartmentalized network (S2_C), agriculturally-intervened non-compartmentalized network (S2_NC). (XLSX)

S16 Table Results for the flux balance analysis. (XLSX)

S17 Table Topological properties of the non-intervened compartmentalized soil sample (S1_C). (XLSX)

S18 Table Topological properties of the intervened compartmentalized soil sample (S2_C). (XLSX)

S19 Table Topological properties of the non-intervened, non-compartmentalized soil sample (S1_NC). (XLSX)

S20 Table Topological properties of the intervened, non-compartmentalized soil sample (S2_NC). (XLSX)
